# Comparison of dural puncture and dural incision in deep brain stimulation surgery: A simple but worthwhile technique modification

**DOI:** 10.3389/fnins.2022.988661

**Published:** 2022-11-03

**Authors:** Shiying Fan, Quan Zhang, Fangang Meng, Huaying Fang, Guang Yang, Zhongjie Shi, Huanguang Liu, Hua Zhang, Anchao Yang, Jianguo Zhang, Lin Shi

**Affiliations:** ^1^Department of Neurosurgery, Beijing Tiantan Hospital, Capital Medical University, Beijing, China; ^2^Department of Functional Neurosurgery, Beijing Neurosurgical Institute, Capital Medical University, Beijing, China; ^3^Beijing Advanced Innovation Center for Imaging Theory and Technology, Capital Normal University, Beijing, China; ^4^Academy for Multidisciplinary Studies, Capital Normal University, Beijing, China; ^5^Department of Neurosurgery, The First Affiliated Hospital of Harbin Medical University, Harbin, China; ^6^Department of Neurosurgery, The First Affiliated Hospital of Xiamen University, Xiamen, China

**Keywords:** dural incision, dural puncture, pneumocephalus, microelectrode recording, electrode displacement

## Abstract

**Background:**

The accuracy of the deep brain stimulation (DBS) electrode placement is influenced by a myriad of factors, among which pneumocephalus and loss of cerebrospinal fluid that occurs with dural opening during the surgery are considered most important. This study aimed to describe an effective method for decreasing pneumocephalus by comparing its clinical efficacy between the two different methods of opening the dura.

**Materials and methods:**

We retrospectively compared two different methods of opening the dura in 108 patients who underwent bilateral DBS surgery in our center. The dural incision group comprised 125 hemispheres (58 bilateral and 9 unilateral) and the dural puncture group comprised 91 (41 bilateral and 9 unilateral). The volume of intracranial air, dural opening time, intraoperative microelectrode recordings (MERs), postoperative electrode displacement, clinical efficacy, and complications were examined. Spearman correlation analysis was employed to identify factors associated with the volume of intracranial air and postoperative electrode displacement.

**Results:**

The volume of intracranial air was significantly lower (0.35 cm^3^ vs. 5.90 cm^3^) and dural opening time was significantly shorter (11s vs. 35s) in the dural puncture group. The volume of intracranial air positively correlated with dural opening time. During surgery, the sensorimotor area was longer (2.47 ± 1.36 mm vs. 1.92 ± 1.42 mm) and MERs were more stable (81.82% vs. 47.73%) in the dural puncture group. Length of the sensorimotor area correlated negatively with the volume of intracranial air. As intracranial air was absorbed after surgery, significant anterior, lateral, and ventral electrode displacement occurred; the differences between the two groups were significant (total electrode displacement, 1.0mm vs. 1.4mm). Electrode displacement correlated positively with the volume of intracranial air. Clinical efficacy was better in the dural puncture group than the dural incision group (52.37% ± 16.18% vs. 43.93% ± 24.50%), although the difference was not significant.

**Conclusion:**

Our data support the hypothesis that opening the dura via puncture rather than incision when performing DBS surgery reduces pneumocephalus, shortens dural opening time, enables longer sensorimotor area and more stable MERs, minimizes postoperative electrode displacement, and may permit a better clinical efficacy.

## Introduction

Successful deep brain stimulation (DBS) surgery depends on accurate placement of implanted electrodes in the pre-planned target area which is determined on specific and dedicated software working with multimodal imaging. Since the surgical planning is based on static preoperative anatomical images, brain shift due to pneumocephalus and loss of cerebrospinal fluid (CSF) during surgery is a well-recognized source of stereotactic inaccuracy (Khan et al., 2008; Obuchi et al., 2008; Petersen et al., 2010). It is believed that brain shift begins to occur as soon as the skull and dura are opened and is at a maximum at the end of surgery. As intracranial air resolves over the days and weeks after surgery, the intraoperative brain shift resolves and the brain returns to its preoperative condition (van den Munckhof et al., 2010; Slotty et al., 2012; Beggio et al., 2020). Thus, the impact of pneumocephalus in DBS surgery should be considered in both periods—during the initial shift as well as the return to the preoperative state (Halpern et al., 2008; Khan et al., 2008; Obuchi et al., 2008; Matias et al., 2018; Piacentino et al., 2021). The brain shift has been extensively studied in previous research, and it is reported that the degree of brain shift correlates with the volume of intracranial air (Matias et al., 2018). However, the impact of pneumocephalus on electrode position has not been thoroughly examined.

Considering its impact on surgical efficacy and its complicated procedure, various techniques for preventing pneumocephalus during DBS procedures have been reported, such as intermittently irrigating the burr hole with saline solution, avoiding CSF suctioning, and sealing dural defects with fibrin glue or other sealant (Petersen et al., 2010; Takumi et al., 2013; Sasaki et al., 2018). Nonetheless, a sufficiently effective technique has not yet been identified.

In this study, we compare two different methods of opening the dura during DBS procedures with regard to the volume of intracranial air, dural opening time, intraoperative microelectrode recordings (MERs), postoperative electrode displacement, and some other aspects. Factors that predict the volume of intracranial air and postoperative electrode displacement are also explored.

## Patients and methods

### Patients

We retrospectively reviewed the clinical data of Parkinson’s disease (PD) patients who underwent bilateral subthalamic nucleus (STN)-DBS surgery in the Department of Neurosurgery at Beijing Tiantan Hospital from September 2020 to August 2021. A total of 180 patients were identified. Patients whose clinical data were incomplete and those lost to follow-up were excluded. Finally, 108 patients (66 males and 42 females, 216 hemispheres) were included for analysis. All patients provided written informed consent. All procedures performed in this study were in accordance with the ethical standards of the ethics committee of Beijing Tiantan Hospital and the Helsinki Declaration (as revised in 2013).

### Surgical procedure

Bilateral STN-DBS electrode implantation was performed under local anesthesia by an experienced functional neurosurgical team using a Leksell microstereotactic system (Elekta Instrument AB, Stockholm, Sweden). In the operation room, the preoperative magnetic resonance imaging (MRI) and computed tomography (CT, thickness: 0.625 mm) data were imported and fused in the Leksell Surgiplan software (Elekta Instruments AB, Stockholm, Sweden). The target and trajectory were determined in the T2 (thickness: 2 mm) and T1(thickness: 1 mm) sequence, respectively. Briefly, all patients were placed in the semi-supine position with the head elevated 25 to 35 degrees. Burr holes were approximately 14 mm in size and placed according to the cortical entry point of the planned trajectory. The dura under the burr hole was opened using one of the two methods described below. MER was conducted with one microelectrode in each hemisphere by the NeuroOmega system (Alpha Omega Engineering, Israel). Electrophysiological signals were recorded to reveal entry into and exit from the STN to guide implantation of the DBS electrodes (3389 Medtronic, Minneapolis, MN). After a standard STN signal was identified, stimulation effectiveness and side effects were tested using a temporary stimulator to assess the optimal location for permanent electrode implantation. Microelectrode trajectories were planned to avoid penetrating the lateral ventricles and sulcus.

**FIGURE 1:**
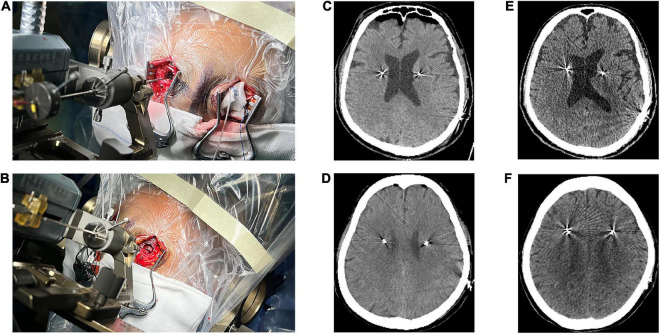
Two different methods of opening the dura. **(A)** Dural incision. **(B)** Dural puncture. **(C)** Pneumocephalus on computed tomography (CT) immediately performed after surgery in a dural incision group patient. **(D)** Pneumocephalus on CT immediately performed after surgery in a dural puncture group patient. **(E)** CT performed one month after surgery in a dural incision group patient showed no pneumocephalus. **(F)** CT performed one month after surgery in a dural puncture group patient showed no pneumocephalus.

Patients and hemispheres were divided into two groups according to dural opening technique. The dural opening technique was randomly selected. In case of dural vascular hemorrhage, blurred puncture field or there were blood vessels beneath the puncture area during dural puncture, dural incision was adopted. The dural incision technique was used in 58 patients, the dural puncture technique in 41, and hybrid technique in 9 (dural incision on one side and dural puncture on the other). Among the 9 patients with hybrid technique, dural incision was adopted due to dural vascular hemorrhage in one patient, blood vessels beneath the puncture area in 3, and blurred puncture field in 5 during the procedure of dural puncture. In the dural incision group, the dura was incised in a cruciate manner. Then, after ensuring no blood vessels were present on the brain surface, the arachnoid was coagulated using a bipolar and incised with a scalpel (Figure 1A). In the dural puncture group, the dura was penetrated by the cannula with the help of monopolar coagulation (Figure 1B). For both types of opening, the burr hole was filled with fibrin glue to avoid CSF leakage.

### Pneumocephalus measurement and dural opening time

The volume of intracranial air was tagged in a slice-by-slice manner on head CT (thickness: 0.625 mm) performed immediately after surgery (3 to 4 h) and one month later using Image J software version 1.31.^1^ Intracranial air collections over both hemispheres of the brain were independently delineated to calculate air volume as follows:


Volume(cm)3=∑S*thickness1000,

where ΣS is the sum of all layers’ air area S (mm^2^).

The volume of intracranial air was independently calculated by two surgeons and averaged. We also studied the difference in volume of intracranial air between the first and the second hemisphere, with patients with hybrid technique excluded. The operation time used to open the dura was also calculated. Dural opening time was defined as the time from the start of dural incision or puncture to the end of cannula insertion.

### Assessment of microelectrode recordings

**FIGURE 2:**
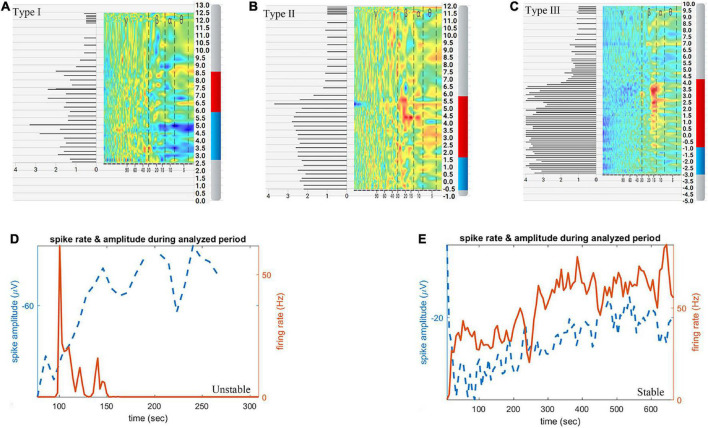
Schematic diagram of subthalamic nucleus (STN) length, length of the sensorimotor area, classification of normalized root mean square (NRMS) and microelectrode recordings (MERs) stability. **(A)** Type I NRMS. **(B)** Type II NRMS. **(C)** Type III NRMS. **(D)** Unstable MERs. **(E)** Stable MERs. The red and blue bars in panels **(A–C)** indicate STN length, while the red bar in panels **(A–C)** indicates length of the sensorimotor area.

During MER, the Haguide module of the NeuroOmega system was used to identify the entry into and exit from the STN as well as the sensorimotor area of STN (Zaidel et al., 2009, 2010). The following MER data were recorded: number of microelectrode tracks, STN length (distance between the STN entry and exit points), length of the sensorimotor area (distance from the sensorimotor area entry to the exit points), and the normalized root mean square (NRMS). We classify the NRMS into 3 categories according to the following criteria: type I is that the NRMS in most parts (>50%) of STN is lower than 2, type III is that the NRMS in most parts (>50%) of STN is higher than 3, others belong to type II, shown in Figures 2A–C.

Aiming to identify the exit of the STN, the signal of the substantia nigra (SNr) was sought for in another 99 hemispheres (44 hemispheres in the dural incision group, 55 in the dural puncture group). Single-unit activity (SUA) was sorted using the open-source semiautomatic template-matching algorithm OSort (Hayase et al., 1998; Faraut et al., 2018). Clusters that represented putative single neurons were identified using criteria described in a previous study, as shown in Supplementary Figure 1 (Faraut et al., 2018). MERs stability was judged by an electrophysiologist according to spike sorting results. MERs stability was categorized as stable or unstable. Unstable MERs indicates that the discharge frequency of neurons gradually decreases to zero Hz over time; that is, the phenomenon of neuron loss occurs, and its duration is less than 10 min (Figure 2D). Stable MERs indicates that the neuronal discharge frequency may fluctuate but will not drop to zero Hz; that is, the neurons continue to discharge and do so for more than 10 min (Figure 2E). The number of single neurons identified per hemisphere and the stability of MERs was analyzed.

### Assessment of electrode location

Postoperative electrode displacement was defined as the difference in stereotactic coordinates of the lowest electrode contact between the immediate and one-month postoperative imaging studies. Co-registration of postoperative CT and preoperative MRI, contact coordinates were performed and obtained using Leksell Surgiplan software. Distances in the X-, Y- and Z-axes were defined as the difference in the mediolateral, anteroposterior, and dorsoventral directions, respectively. Negative values along the X-, Y-, and Z-axes indicated shift to the medial, posterior and ventral direction, meanwhile, the positive values indicate shift to the lateral, anterior and dorsal direction. Calculations were independently performed by two surgeons and then averaged. Total electrode displacement was calculated as follows:


$$\mathrm{Total}\,\mathrm{electrode}\,\mathrm{displacement}\,\left(\mathrm{mm}\right)=\sqrt{{\text{X}}^{2}+{\text{Y}}^{2}+}\,{\text{Z}}^{2}$$

### Clinical assessment and complications

The Unified Parkinson’s Disease Rating Scale Part-III (UPDRS-III) was used to assess motor symptoms in the medication off state before surgery and one month after surgery during stimulation. The medication off state was defined as no levodopa medication administration for at least 12 h. The clinical improvement was computed as follows:


$$\mathrm{Clinical}\,\mathrm{improvement}=\frac{\text{Pre}-\mathrm{scores}-\mathrm{Post}-\text{scores}}{\text{Pre}-\text{scores}}$$

×100%

Perioperative complications such as intraoperative intracranial hemorrhage, intraoperative dural vascular hemorrhage, postoperative intracranial hemorrhage, venous air embolism, pulmonary infection, hallucination, and dyskinesia were recorded. Stimulation-related adverse effects were also assessed.

### Correlation analysis

Spearman correlation analysis was used to identify factors associated with the volume of intracranial air and postoperative electrode displacement. Factors examined included age of onset, age at DBS, course of disease before surgery, blood pressure, dural opening time, number of microelectrode tracks, STN length, length of the sensorimotor area, NRMS, and improvement of med-off UPDRS-III score after surgery.

### Statistical analysis

Patient characteristics are expressed as means with standard deviation or medians with range. Normally distributed data were compared using the independent sample *t*-test or paired sample *t*-test; the Mann–Whitney U test and Wilcoxon signed-rank test were used to compare data with a skewed distribution. Categorical data were compared using the chi-squared test. One-way analysis of variance (ANOVA) was used for comparison of clinical data among three groups. Furthermore, LSD or Tamhane’s T2 was used for post-hoc test. *P* < 0.05 was considered significant. Statistical analyses were performed using IBM SPSS (version 20.0; SPSS Inc, Chicago, IL, USA).

## Results

### Patient characteristics

Patient characteristics are summarized in Table 1. The dural incision group comprised 67 patients (58 bilateral and 9 unilateral, 125 hemispheres) and the dural puncture group comprised 50 (41 bilateral and 9 unilateral, 91 hemispheres). The two groups were similar with respect to gender (*P* = 1.000), age of onset (*P* = 0.074), course of disease before surgery (*P* = 0.712), age at DBS (*P* = 0.053), and med-off UPDRS-III score before surgery (*P* = 0.865).

**TABLE 1:** Patient baseline characteristics by methods of opening the dura.

Variable	Incision group	Puncture group	*P*-value
No. of patients	67	50	–
Male gender – No. (%)	42 (62.69%)	31(62.00%)	1.00
Age of onset (year)	54.97 ± 9.35	51.76 ± 9.72	0.0744
Course of disease before surgery (year)	8.27 ± 4.03	8.56 ± 4.44	0.712
Age at DBS (year)	63.25 ± 7.90	60.32 ± 8.21	0.0531
Med-off UPDRS-III score	50.69 ± 16.20	51.22 ± 17.57	0.865

DBS, deep brain stimulation; Med, medication; UPDRS-III, Unified Parkinson’s Disease Rating Scale Part-III.

Values are presented as mean ± standard deviation (SD).

*P* values for comparisons between groups are based on analysis of independent sample *t*-test and chi-squared test (gender).

As shown in Supplementary Tables 1, 2, the coordinates of the left and right STN in the dural incision group were (110.19 ± 12.63, 98.51 ± 3.47, 105.90 ± 6.72) and (87.45 ± 2.51, 98.87 ± 3.29, 106.19 ± 7.02), respectively. The corresponding coordinates in the dural puncture group were (111.73 ± 2.29, 98.63 ± 3.50, 105.59 ± 7.26) and (88.22 ± 4.53, 98.81 ± 3.58, 104.92 ± 6.77), respectively.

### Pneumocephalus and dural opening time

**FIGURE 3:**
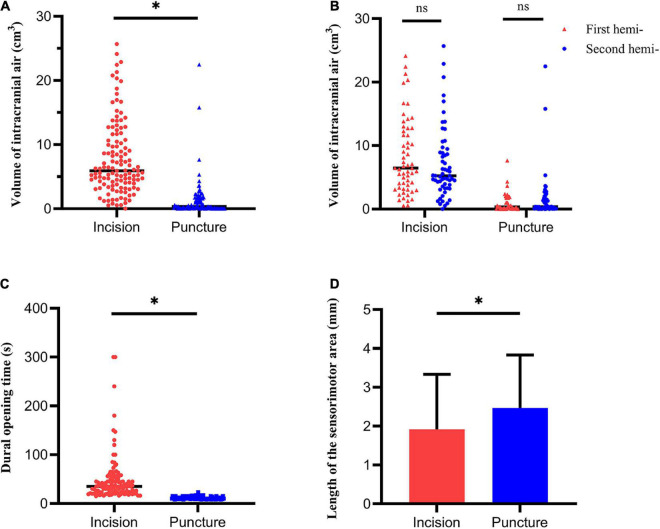
Pneumocephalus, dural opening time and MERs outcomes. **(A)** The volume of intracranial air significantly differed between the dural incision and dural puncture groups. **(B)** The volume of intracranial air showed no significant difference between the first and the second hemisphere. **(C)** Dural opening time significantly differed between the dural incision and dural puncture groups. **(D)** Length of the sensorimotor area significantly differed between the dural incision and dural puncture groups. Hemi-, hemisphere; *P < 0.05; ns, no significance.

The volume of intracranial air on CT performed immediately after surgery was significantly higher in the dural incision group (5.90 cm^3^ [range, 0–25] vs. 0.35 cm^3^ [range, 0–22.49], *P* < 0.001; Figures 1C,D, 3A). There was no significant difference between the unilateral and bilateral incision (*P* = 0.359) or puncture (*P* = 0.372) hemispheres in terms of the volume of intracranial air, observable in Supplementary Table 3. Pneumocephalus was not visible on CT performed one month after surgery in either group (Figures 1E,F). The volume of intracranial air did not significantly differ according to order of hemisphere in which the dura was opened [first vs. second hemisphere; dural incision, 6.46 cm^3^ (range, 0.54–24.13) vs. 5.23 cm^3^ (range, 0–25.68), *P* = 0.238; dural puncture, 0.27 cm^3^ (range, 0–7.63) vs. 0.35 cm^3^ (range, 0–22.49), *P* = 0.197; Figure 3B], with patients with hybrid technique excluded. Dural opening time was significantly longer in the dural incision group [35s (range, 15–300) vs. 11s (range, 8–23), *P* < 0.001; Figure 3C], and no significant difference between the unilateral and bilateral incision (*P* = 0.374) or puncture (*P* = 0.731) hemispheres regarding to dural opening time was noted, shown in Supplementary Table 3.

### Microelectrode recording data

Microelectrode recording (MER) data were available in 118 dural incision group hemispheres and 80 dural puncture group hemispheres. The length of the sensorimotor area was significantly longer in the dural puncture group (2.47 ± 1.36 mm vs. 1.92 ± 1.42 mm, *P* = 0.008; Figure 3D). Number of microelectrode tracks (1.12 ± 0.44 vs. 1.08 ± 0.27, *P* = 0.499), STN length (5.42 ± 1.32 mm vs. 5.49 ± 1.08 mm, *P* = 0.419), and NRMS (2.02 ± 0.75 vs. 2.06 ± 0.70, *P* = 0.667) did not significantly differ between groups. There was also no significant difference between the unilateral and bilateral incision (or puncture) hemispheres in terms of number of microelectrode tracks, STN length, the length of the sensorimotor area, and NRMS, shown in Supplementary Table 3 (all *P* > 0.05).

Number of single neurons per hemisphere did not significantly differ between groups [1 (range, 0–3) vs. 1 (range, 0–2), *P* = 0.135]. The proportion of hemispheres with stable MERs was significantly higher in the dural puncture group (81.82% vs. 47.73%, *P* = 0.001).

### Electrode displacement

**FIGURE 4:**
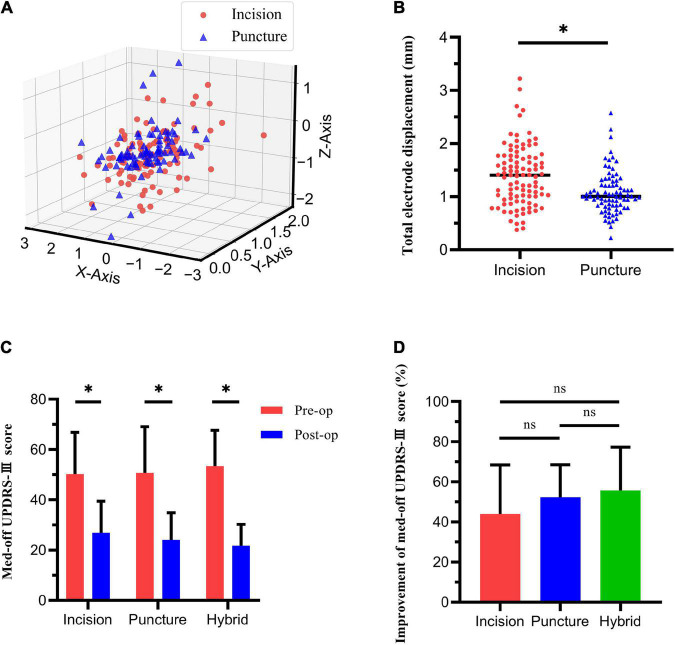
Electrode displacement and change in med-off Unified Parkinson Disease’s Rating Scale Part-III (UPDRS-III) score. **(A)** Electrode displacement in X, Y, and Z-axes in dural incision and dural puncture groups. **(B)** Total electrode displacement significantly differed between the dural incision and dural puncture groups. **(C)** Med-off UPDRS-III score before and after deep brain stimulation surgery in three groups. **(D)** Improvement of med-off UPDRS-III score did not significantly differ among three groups. *P < 0.05; ns, no significance.

Ninety-nine hemispheres in the dural incision group and 81 hemispheres in the dural puncture group underwent CT both immediately and one month after surgery. Significant anterior [0.8mm (range, –0.1 to 2.8), *P* < 0.001; 0.6mm (range, –0.3 to 2.0), *P* < 0.001], lateral [0.3mm (range, –2.9 to 1.7), *P* < 0.001; 0.1mm (range, –0.7 to 1.3), *P* < 0.001], and ventral [–0.6mm (range, –2 to 0.8), *P* < 0.001; –0.5mm (range, –2.1 to 1.2), *P* < 0.001] electrode displacement was seen on the one-month CT scan in both groups (Figure 4A). Detailed coordinates of the lowest electrode contact on the CT scans performed immediately and one month after surgery are provided in Supplementary Tables 4, 5.

Electrode displacement significantly differed between groups in the Y- (*P* = 0.047) and Z- (*P* = 0.008) axes, but not the X-axis (*P* = 0.147). Total electrode displacement was significantly smaller in the dural puncture group [1.0mm (range, 0.22–2.58) vs. 1.4mm (range, 0.37–3.22), *P* < 0.001; Figure 4B]. Besides, there was no significant difference between the unilateral and bilateral incision (or puncture) hemispheres in electrode displacement in the X-axis, Y-axis, Z-axis or the total electrode displacement (all *P* > 0.05), shown in Supplementary Table 3.

### Change of med-off Unified Parkinson’s Disease Rating Scale Part-III score and complications

Since hybrid technique was used in 9 patients, we compared the improvement of med-off UPDRS-III score among the bilateral dural incision group, bilateral dural puncture group and hybrid group. All these three groups exhibited significant improvement of med-off UPDRS-III score after surgery. The bilateral dural incision group score improved from 50.26 ± 16.55 to 26.90 ± 12.56 (*P* < 0.001), the bilateral dural puncture group score from 50.73 ± 18.34 to 24.05 ± 10.76 (*P* < 0.001), and the hybrid group score from 53.44 ± 14.20 to 21.78 ± 8.44 (*P* = 0.001; Figure 4C). There was no significant difference among the three groups in terms of improvement of med-off UPDRS-III score (43.93% ± 24.50% vs. 52.37% ± 16.18% vs. 55.67% ± 21.65%, *P* = 0.090), although the improvement was higher in the bilateral dural puncture group than the bilateral dural incision group (*P* = 0.057; Figure 4D).

Perioperative complications in the dural incision group included dural vascular hemorrhage in 10 patients, hallucination in 3, pulmonary infection in one, and dyskinesia in one. Among the 10 patients with dural vascular hemorrhage in dural incision group, one patient underwent dural incision due to dural vascular hemorrhage during dural puncture. In the dural puncture group, hallucination occurred in two patients, and dyskinesia in one. Stimulation-induced dyskinesia occurred in 18 dural incision group patients and 10 dural puncture group patients. No other stimulation-related side effects were observed.

### Correlation analysis

**FIGURE 5:**
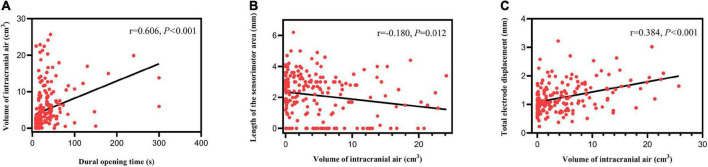
Correlation analysis results. **(A)** The volume of intracranial air correlated positively with dural opening time. **(B)** Length of the sensorimotor area correlated negatively with the volume of intracranial air. **(C)** Total electrode displacement correlated positively with the volume of intracranial air.

The volume of intracranial air was positively correlated with dural opening time (*r* = 0.606, *P* < 0.001; Figure 5A). Length of the sensorimotor area was negatively correlated with the volume of intracranial air (*r* = -0.180, *P* = 0.012; Figure 5B). Anterior (*r* = 0.227, *P* = 0.002), ventral (*r* = 0.232, *P* = 0.002), and total (*r* = 0.384, *P* < 0.001) (Figure 5C) electrode displacements were all positively correlated with the volume of intracranial air. No significant correlation was found between lateral electrode displacement and the volume of intracranial air (*r* = 0.115, *P* = 0.123). Other patient characteristics, including blood pressure, were not significantly correlated with the volume of intracranial air or electrode displacement (all *P* > 0.05). Improvement of med-off UPDRS-III score was not significantly correlated with the volume of intracranial air or electrode displacement (all *P* > 0.05).

## Discussion

In this retrospective study of patients who underwent bilateral STN-DBS for treatment of PD, the volume of intracranial air was significantly lower in patients whose dura was opened via puncture compared to those whose dura was opened via incision (5.90 cm^3^ vs. 0.35 cm^3^). Moreover, dural opening time was shorter (35 s vs. 11 s) in the dural puncture group. The length of the sensorimotor area was longer, the proportion of hemispheres with stable MERs was significantly higher and electrode displacement was significantly lower in the dural puncture group. Besides, dural puncture group showed better improvement of med-off UPDRS-III score than the dural incision group, although the difference was not significant. We also found that the volume of intracranial air correlated negatively with length of the sensorimotor area and positively correlated with dural opening time and electrode displacement. To the best of our knowledge, this is the first comprehensive comparison of clinical efficacy between dural puncture and dural incision, including not only pneumocephalus, but also dural opening time, intraoperative MERs, especially MERs stability, postoperative electrode displacement, and clinical efficacy.

During surgery, air is introduced into the intracranial cavity immediately after dural opening and is hypothesized to be at its maximum at the time of surgery (Slotty et al., 2012; Beggio et al., 2020). The cerebral cortex and deep nuclei have already shifted even before electrodes have been placed. Therefore, the position of the final electrode implantation relative to STN will change, with possible therapeutic implications. The brain shift is generally in a posterior direction and ranges from 0 to 10.1 mm (Miyagi et al., 2007; Halpern et al., 2008; Khan et al., 2008; Obuchi et al., 2008; Ko et al., 2018). The greatest degree of shift occurs in the frontal lobe, followed by the temporal and occipital lobes. Shift is also observed in deep structures such as the anterior and posterior commissures and basal ganglia. Although shift in the pallidum and subthalamic region ipsilateral to the burr hole averages 0.5 to 1 mm, 9% of patients exhibit >2 mm of shift in deep brain structures; some exhibit shift up to 4 mm (Sillay et al., 2013).

To enable accurate electrode implantation during DBS surgery, intraoperative MER can be used to adjust for brain shift (Halpern et al., 2008; Petersen et al., 2010). Previous studies have reported that brain shift is associated with number of microelectrode tracks made during surgery (Azmi et al., 2011; Sasaki et al., 2018); however, volume of intracranial air and number of microelectrode tracks have not been definitively linked. In the present study, we observed a significant difference in length of the sensorimotor area between the dural puncture and incision groups (2.47mm vs. 1.92mm) and found that the length of the sensorimotor area correlated negatively with the volume of intracranial air. Furthermore, MERs were more stable in the dural puncture group, which may be due to a more stable condition of CSF, and it was conducive to the accurate implantation of electrode. Overall, our findings confirmed that pneumocephalus affects electrode position during DBS surgery and that MER can guide accurate electrode implantation. Dural puncture can reduce pneumocephalus, and enable longer sensorimotor area and more stable MERs, which guide accurate electrode implantation.

In our study, the electrode shifted anteriorly, laterally, and ventrally during the intracranial air absorption period after surgery, and the shift was big enough to change electrode position relative to STN. Furthermore, electrode displacement correlated with the volume of intracranial air. These results are consistent with previous studies that reported DBS electrode position can change on delayed postoperative imaging and that this change is significantly correlated with the volume of intracranial air (van den Munckhof et al., 2010; Göransson et al., 2021). Direction of electrode displacement in our study was consistent with a previous study (Göransson et al., 2021). However, another study found that electrode displacement tended to be upward; the authors therefore advocated slightly deeper initial electrode placement (van den Munckhof et al., 2010). The reasons for the inconsistent results in different studies may be that on the one hand, different researchers adopt different methods to locate electrode contacts in postoperative CT, on the other hand, a variety of interference factors such as imaging distortion, error registration, and mechanical frame inaccuracy may have a certain impact on the research results in different studies. Thus, direction of electrode displacement warrants further investigation. It is also worth noting that in this study, the difference in electrode displacement between the two groups as well as the positive correlation between electrode displacement and the volume of intracranial air was only noted in the Y- and Z-axes, which may imply that the influence of pneumocephalus on electrode displacement is in the Y- and Z-axes. It still needs further confirmation. Regardless, our study and the studies of others indicate that postoperative electrode displacement occurs. We strongly advocate repeated postoperative imaging after DBS surgery to monitor electrode location, especially in patients who show considerable volume of intracranial air on immediate postoperative imaging (Kim et al., 2010). Furthermore, we advocate opening the dura via puncture rather than incision, as puncture is associated with a lower degree of electrode displacement.

The dural puncture group showed better improvement of med-off UPDRS-III score than the dural incision group (52.37% vs. 43.93%), although the difference was not significant. Improvement of med-off UPDRS-III score was not significantly correlated with electrode displacement or the volume of intracranial air. Also, in the dural puncture group, dural opening time was significantly shorter (11 min vs. 35 min) for its simple operation procedure and lower risk of dural vascular hemorrhage. Dural vascular hemorrhage can account for the significant longer dural opening time in several hemispheres, especially in the dural incision group. In the present study, the incidence of dural vascular hemorrhage in the dural incision group was higher than that in the dural puncture group. It may be due to the limited subdural field of vision during incision and the large scope of dural damage, while dural puncture causes less damage to the dura and monopolar coagulation has a certain hemostatic effect. Besides, unpredictable variations of the vessel position caused by larger brain shift in dural incision group may account for its higher risk of dural vascular hemorrhage, which may be confirmed by pre and postoperative contrast-enhanced MRI or CT in future study. It should be noted that in this study, one patient underwent dural incision due to dural vascular hemorrhage during dural puncture. We classified it as the dural incision group, but it was actually a complication of dural puncture. Despite all this, the incidence of dural vascular hemorrhage was still higher in the dural incision group. Dural puncture may cause less damage to the cerebral cortex for its minimal dural incision; however, it may also limit inspection of the brain parenchyma and brain surface, which may increase the risk of intracranial hemorrhage. This risk may be reduced by paying close attention to cortical vessels when planning the trajectory in MRI, especially in contrast-enhanced MRI. In case of dural vascular hemorrhage, blurred puncture field or there were blood vessels beneath the puncture area during dural puncture, it may be necessary to resort to dural incision. Dural incision may be more suitable for multichannel MER.

Several factors influence the volume of intracranial air introduced during DBS surgery: brain volume, patient position, duration of surgery, burr hole localization, size of opening in the CSF space, blood pressure, and method of burr hole closure (Slotty et al., 2012). Preoperative cerebral atrophy may be related to increased presence of pneumocephalus (Piacentino et al., 2021). Strict supine positioning during surgery is associated with minimal volume of intracranial air. Previous studies have demonstrated that CSF egress and air entry are immediate results of dural opening. CSF loss later during surgery can be effectively averted by burr hole closure (Piacentino et al., 2021). Consequently, shorter surgeries have not been associated with target precision or DBS clinical outcome. However, we found a significant positive correlation between dural opening time and the volume of intracranial air in the present study. These contradictory results may be due to the different definitions of operation time in different studies. Smaller burr hole diameter has not been associated with less air inflow or displacement of anatomical targets (Sharim et al., 2015). Lower systolic and mean arterial blood pressure have been associated with higher volume of intracranial air (Krauss et al., 2021). Moreover, significantly greater brain shift has been reported in patients with intraparenchymal trajectories (Kramer et al., 2012). Compared to patients undergoing awake DBS, lower volume of intracranial air has been reported in patients undergoing DBS under general anesthesia (Ko et al., 2018). Electrode texture can also influence electrode displacement: one previous study reported that electrode migration distance is lower with microtextured electrodes (Parittotokkaporn et al., 2012).

Given that the degree of brain shift correlates with the volume of intracranial air, a number of surgical practices can be expected to reduce or avoid brain shift. CSF loss and pneumocephalus can be reduced by minimizing the time from dural opening to final DBS electrode implantation, flooding the burr hole with saline irrigation after dural opening, avoiding CSF suction, and sealing the dural defect as soon as possible (Petersen et al., 2010; Takumi et al., 2013; Sasaki et al., 2018). Performing surgery with the patient in the same position as when imaging was acquired may also limit shift by minimizing postural movement of intracranial structures. Although the sitting or semi-sitting position can decrease CSF leakage, both are associated with increased risk of venous air embolism (Nazzaro et al., 2010; Petersen et al., 2010; Takumi et al., 2013). Patient positioning can be difficult with PD patients, as many have postural abnormalities that must be considered and dealt with. Although various techniques to deal with pneumocephalus have been reported, an effective one has not yet been identified.

In the present study, all patients underwent surgery using local anesthesia in a fixed semi-supine position with head elevation fixed between 25 and 35 degrees. Burr holes were approximately 14 mm in size and were placed 30 to 35 mm anterior to the bregma. Besides, none of the planned microelectrode trajectories penetrated the lateral ventricles. Although the baseline age of onset and age at DBS of the two groups were different, correlation analysis showed no significant correlation between them and the volume of intracranial air or postoperative electrode displacement. None of other patient characteristics, including blood pressure, was associated with the volume of intracranial air or postoperative electrode displacement in our study. Furthermore, among the 9 patients using hybrid technique, our results showed no significant difference between bilateral and unilateral incision (or puncture) hemispheres. Thus, the results of the comparison between the two groups in our study were credible. Recording of local field potentials, macrostimulation, and clinical testing through the implanted DBS electrode probably extend the operation time in procedures performed under local anesthesia. However, these activities were performed after the dural defect was sealed with fibrin glue. The fact that the volume of intracranial air was similar between the first and the second hemisphere confirmed the effectiveness of our method of dural closure.

Our study has several limitations. First, it was conducted retrospectively. Second, MRI was not performed after surgery, which limited the calculation of brain shift. To determine brain shift in real time during surgery, intraoperative ultrasound and/or MRI is considered promising.

## Conclusion

Opening the dura via puncture rather than incision when performing DBS surgery reduces pneumocephalus, shortens dural opening time, enables longer sensorimotor area and more stable MERs, minimizes postoperative electrode displacement, and may permit a better clinical efficacy.

## Data availability statement

The raw data supporting the conclusions of this article will be made available by the authors, without undue reservation.

## Ethics statement

The studies involving human participants were reviewed and approved by the Ethics Committee of Beijing Tiantan Hospital. The patients/participants provided their written informed consent to participate in this study.

## Author contributions

SF, LS, JZ, and QZ made the conception and design. SF, QZ, and FM contributed to data acquisition and statistical analysis. SF, LS, HF, GY, ZS, HL, and HZ contributed to analysis and interpretation of data. SF and QZ drafted and finalized the manuscript. LS, AY, and JZ critically revised the article. JZ contributed to study supervision. All authors reviewed the submitted version of the manuscript.

## Footnotes

1
http://rsbweb.nih.gov/ij
