# Supplementary material for: Comparison of dural puncture and dural incision in deep brain stimulation surgery: A simple but worthwhile technique modification
Source: Front Neurosci. 2022 Nov 3;16:988661. doi: 10.3389/fnins.2022.988661 (PMC9669717; doi:10.3389/fnins.2022.988661)
Supplement: Supplementary file 1 [file Data_Sheet_1.docx]

Supplementary Material

**Supplementary Table 1. Coordinates of STN in dural incision group**

| **Patients** | **Left hemisphere** | | | **Right hemisphere** | | |
| --- | --- | --- | --- | --- | --- | --- |
|  | **X** | **Y** | **Z** | **X** | **Y** | **Z** |
| **1** | 110.8 | 103.7 | 110.9 | 85.3 | 103 | 109.9 |
| **2** | 111 | 92 | 107.5 | 88 | 91.5 | 106.5 |
| **3** | 110 | 99.5 | 115.5 | - | - | - |
| **4** | 114 | 99.5 | 113 | 89 | 100 | 112.1 |
| **5** | 110.5 | 96.5 | 97.8 | 88 | 98 | 97 |
| **6** | 114.2 | 94.8 | 90.7 | 91.2 | 96.2 | 90.9 |
| **7** | 113 | 98.7 | 91.5 | 87.5 | 98.2 | 91.6 |
| **8** | 113.5 | 97 | 99.5 | 88 | 98 | 98.6 |
| **9** | 113.5 | 97 | 118.3 | 88.5 | 98 | 119.2 |
| **10** | 110.5 | 93.5 | 120.2 | 88.5 | 93.5 | 119.7 |
| **11** | 112.6 | 97.5 | 111 | 89.6 | 98 | 110.4 |
| **12** | 112 | 103 | 105.5 | 83.5 | 105.1 | 105.2 |
| **13** | 114 | 103.5 | 106.8 | 85.5 | 102.5 | 105.3 |
| **14** | 110.5 | 94.5 | 100.5 | 87.5 | 96.4 | 101.7 |
| **15** | 110.2 | 104.7 | 107.5 | 85 | 104.6 | 108.1 |
| **16** | 110.5 | 99 | 105.7 | 89 | 100 | 105.8 |
| **17** | 109.5 | 100 | 113.3 | 84 | 101.5 | 113.7 |
| **18** | 109.5 | 100.5 | 111.7 | 88 | 101.5 | 112 |
| **19** | 116.9 | 103 | 100.2 | 89 | 101.5 | 99.5 |
| **20** | 116 | 94 | 104.1 | 90.5 | 93.5 | 104.3 |
| **21** | 116 | 99.3 | 105.9 | 93.8 | 99 | 104.5 |
| **22** | 106 | 97 | 103.4 | 82.7 | 97.5 | 104.6 |
| **23** | 110 | 101.1 | 105.9 | 84.5 | 101.5 | 106.9 |
| **24** | 112.5 | 101.5 | 102.8 | 89 | 102.5 | 103 |
| **25** | 106.5 | 94.2 | 103.3 | 87.9 | 95.5 | 102.6 |
| **26** | 113 | 96.1 | 114 | 89.5 | 96.5 | 113.2 |
| **27** | 111.5 | 100 | 106.3 | 87 | 100.5 | 105.7 |
| **28** | 111 | 97.5 | 97.5 | - | - | - |
| **29** | 112 | 102 | 103.7 | - | - | - |
| **30** | 112 | 94.5 | 103.4 | 84.5 | 94.9 | 102.6 |
| **31** | 107.5 | 98.5 | 121.8 | 81.9 | 99.5 | 119.7 |
| **32** | 111.5 | 94.5 | 104 | - | - | - |
| **33** | 110.1 | 94.6 | 111.3 | - | - | - |
| **34** | 111 | 102.5 | 99.1 | 89.5 | 103.6 | 100 |
| **35** | 112 | 95.5 | 115 | 90.5 | 94.5 | 114.8 |
| **36** | 112.5 | 100 | 118.1 | 90.5 | 101.5 | 118 |
| **37** | 109 | 99 | 99.2 | 86.1 | 100.5 | 98.8 |
| **38** | - | - | - | 83 | 99.9 | 108 |
| **39** | 105 | 98 | 99.5 | 80 | 97.5 | 98.5 |
| **40** | 110.5 | 106 | 101.2 | 87.7 | 103.2 | 102.8 |
| **41** | 110.6 | 99 | 108 | 86 | 97.5 | 108.2 |
| **42** | 111.5 | 99.5 | 101.4 | 88 | 100 | 101 |
| **43** | 116 | 103.5 | 105.8 | 88 | 103.5 | 107.4 |
| **44** | 114 | 96.1 | 106.3 | 88.5 | 96 | 106.8 |
| **45** | 113 | 97.5 | 108.9 | 88.5 | 96.9 | 107.1 |
| **46** | 112.8 | 99.3 | 93.3 | 85.4 | 97.3 | 94 |
| **47** | 108.4 | 98.2 | 121 | 85.2 | 97.9 | 120.4 |
| **48** | 113 | 94 | 102.4 | 90.5 | 96 | 102.6 |
| **49** | - | - | - | 88 | 97.1 | 125 |
| **50** | 109.5 | 94.4 | 103.2 | 88 | 95 | 105.2 |
| **51** | 112 | 96.9 | 118.1 | 86.5 | 96.5 | 117.3 |
| **52** | 113 | 93.9 | 103.7 | - | - | - |
| **53** | 111 | 97.4 | 113.4 | 89 | 98 | 112.2 |
| **54** | 109 | 94.5 | 104.9 | 86.5 | 94.5 | 104.5 |
| **55** | 110.5 | 100 | 100.1 | 86.5 | 101.5 | 99 |
| **56** | 112.2 | 96.5 | 105 | 89 | 96 | 105 |
| **57** | 114.5 | 102.1 | 103.5 | 89 | 101.5 | 104.3 |
| **58** | 112 | 97 | 104.7 | 85 | 98.5 | 104.5 |
| **59** | 109.5 | 96 | 106.9 | 87 | 98 | 106.9 |
| **60** | 110.5 | 94 | 103 | 86 | 94.5 | 103 |
| **61** | 115.5 | 107.5 | 101.7 | 89.6 | 105 | 103.9 |
| **62** | 114.1 | 95.4 | 105.4 | 87 | 94 | 105.5 |
| **63** | 111 | 104 | 104.4 | 90.1 | 104.5 | 106.6 |
| **64** | 115 | 102 | 98.2 | 91 | 101 | 97 |
| **65** | 115.5 | 98 | 103.5 | - | - | - |
| **66** | 114.2 | 103.2 | 106 | 89.2 | 103.6 | 105.1 |
| **67** | 111 | 99.5 | 104.1 | 85.5 | 98.5 | 103.9 |

Abbreviations: STN, subthalamic nucleus.

**Supplementary Table 2. Coordinates of STN in dural puncture group**

| **Patients** | **Left hemisphere** | | | **Right hemisphere** | | |
| --- | --- | --- | --- | --- | --- | --- |
|  | **X** | **Y** | **Z** | **X** | **Y** | **Z** |
| **1** | 114 | 97.5 | 97.4 | 89.6 | 98.5 | 96.1 |
| **2** | 116 | 104.2 | 115.3 | 90 | 103.7 | 115.8 |
| **3** | - | - | - | 86.5 | 101.5 | 115 |
| **4** | 111 | 98.6 | 108.2 | 86.8 | 99.9 | 107.5 |
| **5** | 112.5 | 104 | 91.6 | 87 | 104.5 | 89.8 |
| **6** | 112 | 93 | 106.5 | 88 | 93 | 107 |
| **7** | 113 | 100.7 | 108.6 | 88.6 | 102 | 107.1 |
| **8** | 109.3 | 105.6 | 118 | 83 | 105 | 118.2 |
| **9** | 111 | 94 | 105.6 | 88 | 93.9 | 104.5 |
| **10** | 112.7 | 98 | 110.7 | 88.3 | 98 | 110.5 |
| **11** | 113.9 | 99.5 | 105.3 | 87.5 | 99.5 | 104.1 |
| **12** | 110.3 | 98.9 | 107.8 | 87.5 | 96 | 109 |
| **13** | 110.5 | 95.5 | 105.5 | 89.5 | 97.4 | 103.1 |
| **14** | 111.5 | 95 | 112.1 | 91 | 94.2 | 111.9 |
| **15** | 106 | 98 | 107.5 | 84.5 | 99 | 107.5 |
| **16** | 110.6 | 95.9 | 107.3 | 87.2 | 98 | 104.6 |
| **17** | 110.5 | 94.5 | 94.2 | 87.5 | 95 | 94.7 |
| **18** | 110.5 | 94.5 | 94.2 | 87.5 | 95 | 94.7 |
| **19** | 109.5 | 99.4 | 103.5 | 86.5 | 98.4 | 103.5 |
| **20** | 111.5 | 98.9 | 99.7 | 87 | 100 | 100 |
| **21** | 109.5 | 107 | 119.1 | 86.5 | 108 | 119.3 |
| **22** | 111.1 | 99.9 | 103.8 | 89.2 | 99.7 | 103 |
| **23** | 110.1 | 98.9 | 102.5 | 84.1 | 99.2 | 100.1 |
| **24** | 113.1 | 93.5 | 105.5 | 89.5 | 94.5 | 104.9 |
| **25** | 113.5 | 97 | 103.8 | 90.5 | 98 | 103.3 |
| **26** | 115 | 104.5 | 106.4 | 90.3 | 105.9 | 105.4 |
| **27** | 113 | 98.5 | 108.6 | 88 | 98 | 107.5 |
| **28** | - | - | - | 87 | 97.5 | 97 |
| **29** | - | - | - | 89 | 102.1 | 103.3 |
| **30** | - | - | - | 87.5 | 95.5 | 103.5 |
| **31** | - | - | - | 86.4 | 95.7 | 111.2 |
| **32** | 111.5 | 96 | 104.5 | 89 | 96.5 | 103.5 |
| **33** | 112.1 | 101.6 | 109 | - | - | - |
| **34** | 109.5 | 102.5 | 101.5 | 88.5 | 102 | 101.5 |
| **35** | 108.3 | 100.3 | 108.4 | 83.8 | 101.1 | 109.3 |
| **36** | 115.2 | 93 | 105.4 | 88.4 | 93.9 | 106.2 |
| **37** | 115 | 94 | 106 | 92 | 93.5 | 105 |
| **38** | 108 | 100 | 109.6 | 84.5 | 101 | 109.9 |
| **39** | 111 | 100.5 | 95.5 | 87 | 100 | 95.3 |
| **40** | 110 | 102.5 | 98 | 85 | 102.6 | 98.4 |
| **41** | 110.5 | 97 | 118.7 | 88 | 96.5 | 118.7 |
| **42** | 116.5 | 94.5 | 103.9 | 91 | 95.5 | 104 |
| **43** | 113 | 96.6 | 126.6 | - | - | - |
| **44** | - | - | - | 87 | 94 | 102.3 |
| **45** | 108 | 97.5 | 108 | 85.5 | 96.5 | 107.2 |
| **46** | 114 | 98.5 | 100.2 | 88.5 | 99.5 | 99.6 |
| **47** | 114 | 101 | 101.7 | 87 | 100.5 | 100.7 |
| **48** | 113.5 | 98.9 | 103.7 | 85.5 | 97.5 | 105 |
| **49** | 112.5 | 101.5 | 90.9 | 88.3 | 101.5 | 91 |
| **50** | - | - | - | 116 | 104.2 | 115.3 |

Abbreviations: STN, subthalamic nucleus.

**Supplementary Table 3. Comparison between bilateral and unilateral dural incision or puncture hemispheres**

| **Variables** | |  | **Dural incision** | ***P* value** | **Dural puncture** | ***P* value** |
| --- | --- | --- | --- | --- | --- | --- |
| **Pneumocephalus (cm^3^)** | | Bilateral | 5.9[0-25.68] | 0.359 | 0.33[0-22.49] | 0.372 |
|  |  | Unilateral | 7.32[4.75-18.74] |  | 0.97[0-2.18] |  |
| **Dural opening time (s)** | | Bilateral | 36[15-300] | 0.374 | 11[8-23] | 0.731 |
|  |  | Unilateral | 30[19-65] |  | 10[8-17] |  |
| **Microelectrode recording** | **Number of microelectrode tracks** | Bilateral | 1[1-4] | 0.407 | 1[1-2] | 0.412 |
|  |  | Unilateral | 1[1-1] |  | 1[1-1] |  |
|  | **Subtalamic nucleus length (mm)** | Bilateral | 5.5[0-9.5] | 0.740 | 5.4[2.8-8.6] | 0.125 |
|  |  | Unilateral | 5.55[4.7-8.0] |  | 5.65[5.1-8.1] |  |
|  | **The length of the sensorimotor area (mm)** | Bilateral | 1.9[0-5] | 0.200 | 2.65[0-4.2] | 0.216 |
|  |  | Unilateral | 2.6[0-4.4] |  | 3[1-6.2] |  |
|  | **NRMS** | Bilateral | 2[1-3] | 0.671 | 2[1-3] | 0.772 |
|  |  | Unilateral | 2[1-3] |  | 2[1-3] |  |
| **Electrode displacement (mm)** | **X-Axis** | Bilateral | 0.3[-2.9-1.7] | 0.879 | 0.15[-0.7-1.3] | 0.552 |
|  |  | Unilateral | 0.3[-0.3-1.1] |  | 0.1[-0.4-0.7] |  |
|  | **Y-Axis** | Bilateral | 0.8[-0.1-2.8] | 0.617 | 0.6[-0.3-2.0] | 0.629 |
|  |  | Unilateral | 0.6[0.1-1.4] |  | 0.6[0-1.7] |  |
|  | **Z-Axis** | Bilateral | -0.6[-2.0-0.8] | 0.855 | -0.5[-2.1-1.2] | 0.629 |
|  |  | Unilateral | -0.7[-1.0-(-0.4)] |  | -0.5[-0.9-0.2] |  |
|  | **Total** | Bilateral | 1.41[0.37-3.22] | 0.179 | 1.02[0.22-2.58] | 0.994 |
|  |  | Unilateral | 1.05[0.78-1.80] |  | 1.0[0.54-1.85] |  |
| Values are presented as median with range.  *P* values for comparisons between groups are based on Mann–Whitney U test. | | | | | | |

**Supplementary Table 4. Coordinates of the lowest electrode contact in dural incision group**

| **Patient** | **Immediate CT** | | | | | | **One-month postoperative CT** | | | | | |
| --- | --- | --- | --- | --- | --- | --- | --- | --- | --- | --- | --- | --- |
|  | **Left hemisphere** | | | **Right hemisphere** | | | **Left hemisphere** | | | **Right hemisphere** | | |
|  | **X** | **Y** | **Z** | **X** | **Y** | **Z** | **X** | **Y** | **Z** | **X** | **Y** | **Z** |
| 1 | 110.6 | 102.9 | 110.1 | 85.5 | 102.2 | 110.1 | 110.8 | 103.1 | 110.5 | 85.3 | 102.3 | 111.1 |
| 2 | 109 | 91.5 | 107.7 | 87.1 | 90.1 | 106.7 | 109.9 | 92.1 | 107.9 | 87.2 | 90.8 | 106.9 |
| 3 | 109.5 | 98.3 | 116.8 | - | - | - | 110 | 98.9 | 117.6 | - | - | - |
| 4 | 113.5 | 97.6 | 113.8 | 89 | 99 | 111.6 | 114 | 99.1 | 114 | 89 | 100 | 111.7 |
| 5 | 108.1 | 94.6 | 98.9 | 88 | 95.6 | 98.4 | 108.5 | 96.1 | 99.9 | 88.1 | 97.2 | 98.7 |
| 6 | - | - | - | - | - | - | - | - | - | - | - | - |
| 7 | 111.9 | 96.4 | 92.7 | 88.4 | 95.4 | 92.6 | 111.8 | 97.3 | 93.8 | 87.3 | 96.1 | 93.5 |
| 8 | 111.4 | 96.2 | 100.3 | 87.8 | 94.5 | 101.6 | 111.9 | 96.2 | 101.2 | 87.8 | 95.1 | 102.1 |
| 9 | - | - | - | - | - | - | - | - | - | - | - | - |
| 10 | 110.2 | 93.3 | 119.7 | 88.5 | 93.5 | 119.7 | 110.5 | 93.9 | 120 | 87.9 | 94 | 121 |
| 11 | 111.3 | 96.5 | 110.9 | 89.4 | 95.9 | 112.7 | 111.3 | 96.5 | 110.5 | 89.7 | 97.7 | 111.9 |
| 12 | - | - | - | - | - | - | - | - | - | - | - | - |
| 13 | - | - | - | - | - | - | - | - | - | - | - | - |
| 14 | 110.6 | 93.9 | 100.9 | 88.8 | 96.2 | 102.4 | 111.8 | 94.8 | 102.3 | 89.2 | 96.4 | 104.1 |
| 15 | 109.7 | 103.2 | 106.4 | 88 | 103.1 | 109.2 | 109.9 | 104 | 107.9 | 87.3 | 104.1 | 109.8 |
| 16 | 108.7 | 97.1 | 106 | 90.9 | 97.2 | 104.8 | 108.6 | 97.6 | 107 | 90.4 | 97.9 | 106.8 |
| 17 | 107.9 | 98.8 | 113.2 | 85.1 | 99.5 | 113.6 | 105 | 100.2 | 113.3 | 83.8 | 100 | 114.3 |
| 18 | 108.2 | 98.9 | 112.1 | 88.3 | 102.2 | 111.3 | 108.2 | 99.9 | 112.4 | 87.5 | 103.1 | 112.3 |
| 19 | 115.7 | 101.5 | 100.3 | 88.8 | 100.6 | 99.5 | 116.1 | 102 | 101.4 | 88.7 | 101.7 | 100 |
| 20 | 114.9 | 92.3 | 106.6 | 91 | 90.6 | 107.4 | 114.8 | 92.6 | 107.3 | 90.9 | 91.2 | 107.1 |
| 21 | 114 | 96.6 | 107.5 | 93.5 | 97.4 | 105.2 | 114.5 | 96.9 | 108.1 | 93.5 | 97.9 | 106.6 |
| 22 | 105.7 | 97.2 | 102.4 | 81.6 | 97.3 | 105 | 106.4 | 98.9 | 103.1 | 81.6 | 98.6 | 106.5 |
| 23 | 108.8 | 100.1 | 106.6 | 84.6 | 100.1 | 107.2 | 108.7 | 100.6 | 107.2 | 83.6 | 100.8 | 107.9 |
| 24 | - | - | - | - | - | - | - | - | - | - | - | - |
| 25 | - | - | - | - | - | - | - | - | - | - | - | - |
| 26 | 112.9 | 95.4 | 113.5 | 89.3 | 96.5 | 114 | 113.4 | 96.1 | 113.8 | 89 | 97.4 | 114.4 |
| 27 | 109.1 | 97.4 | 107.5 | 87.6 | 96.6 | 109.7 | 109.5 | 98.9 | 109.5 | 87.6 | 98.3 | 109.2 |
| 28 | 109.2 | 96.3 | 98.4 | - | - | - | 109.5 | 97.1 | 98.8 | - | - | - |
| 29 | 110.6 | 101 | 103.5 | - | - | - | 110.7 | 101.3 | 104.5 | - | - | - |
| 30 | - | - | - | - | - | - | - | - | - | - | - | - |
| 31 | 105.7 | 98.4 | 122.1 | 81.7 | 99.3 | 120.7 | 107.2 | 99.9 | 121.7 | 81.6 | 101 | 120.4 |
| 32 | 109.4 | 93.1 | 104.7 | - | - | - | 109.7 | 93.7 | 105.1 | - | - | - |
| 33 | 107.3 | 90.4 | 113.9 | - | - | - | 107.6 | 91.8 | 114.5 | - | - | - |
| 34 | 108.9 | 100.7 | 100.2 | 89.2 | 101.4 | 101.7 | 109.3 | 101.8 | 101.1 | 89.3 | 102.3 | 102.3 |
| 35 | 112.1 | 94.5 | 113.6 | 90.5 | 94.4 | 115.5 | 112.3 | 95.7 | 114.7 | 90 | 95.2 | 116.7 |
| 36 | 112.6 | 100.6 | 116.7 | 90.1 | 100.3 | 118.6 | 112 | 101.3 | 117.1 | 89.1 | 100.8 | 118.9 |
| 37 | 107.5 | 99.1 | 99.5 | 86.1 | 100.5 | 99.3 | 107.2 | 99.9 | 99.7 | 85.4 | 100.9 | 100.2 |
| 38 | 82.8 | 99.3 | 108.6 | - | - | - | 83.1 | 99.8 | 109.3 | - | - | - |
| 39 | 103 | 97.2 | 99.4 | 80 | 96.9 | 98.9 | 104.2 | 97.1 | 100.2 | 80.7 | 97.3 | 100.2 |
| 40 | 109.2 | 104 | 102.2 | 85.4 | 104 | 102.8 | 109 | 104.5 | 104 | 85 | 105.5 | 103.9 |
| 41 | 110 | 98.4 | 107.5 | 85.9 | 95.6 | 109.5 | 109.6 | 98.6 | 108.8 | 85.2 | 97.2 | 110.4 |
| 42 | - | - | - | - | - | - | - | - | - | - | - | - |
| 43 | - | - | - | - | - | - | - | - | - | - | - | - |
| 44 | - | - | - | - | - | - | - | - | - | - | - | - |
| 45 | 112.3 | 95.8 | 109.1 | 88.1 | 95.6 | 108.5 | 112.7 | 96.2 | 109.4 | 87.7 | 95.9 | 108.4 |
| 46 | 109.5 | 99.8 | 94 | 85.8 | 98 | 93.7 | 109.1 | 100.1 | 94.6 | 84.8 | 98.4 | 94.3 |
| 47 | 107.8 | 98.1 | 121.2 | 85.6 | 95.1 | 123.9 | 108.7 | 99.3 | 122.6 | 86.3 | 96.7 | 124.2 |
| 48 | 110.3 | 91.8 | 104.2 | 89.5 | 95.7 | 102.1 | 108.6 | 93 | 104 | 87.8 | 95.8 | 102.6 |
| 49 | 89.1 | 95.3 | 124.9 | - | - | - | 88.5 | 95.4 | 125.5 | - | - | - |
| 50 | 107 | 91.9 | 105.3 | 89 | 92.1 | 107 | 108.1 | 91.9 | 106.9 | 89.9 | 93 | 108.2 |
| 51 | - | - | - | - | - | - | - | - | - | - | - | - |
| 52 | 112.2 | 92.7 | 103.6 | - | - | - | 112.2 | 93.9 | 104.3 | - | - | - |
| 53 | 110.1 | 95.7 | 114.8 | 89.2 | 96.2 | 113.5 | 110.7 | 96.4 | 114.5 | 89.1 | 97.2 | 113.6 |
| 54 | 106.8 | 93.4 | 104.6 | 85.9 | 95.2 | 104.5 | 106.4 | 94.4 | 105.5 | 84.8 | 96.1 | 105 |
| 55 | 107.8 | 97.8 | 99.2 | 86.8 | 101 | 96.8 | 107.8 | 99.9 | 100.9 | 85.7 | 103.8 | 97.1 |
| 56 | 110.1 | 95.1 | 106 | 88.4 | 95.1 | 104.6 | 110.9 | 96.1 | 107.1 | 87.8 | 96 | 105.5 |
| 57 | 113.6 | 101 | 104.7 | 89.8 | 100.5 | 104.3 | 113.5 | 101.3 | 104.5 | 89.3 | 101.1 | 105.2 |
| 58 | 110.4 | 94.8 | 105.2 | 85 | 96.6 | 105.9 | 111.8 | 96 | 106.4 | 85.8 | 97.9 | 106.5 |
| 59 | 107.8 | 93.7 | 105.6 | 87.4 | 95.4 | 106.4 | 108.1 | 94.8 | 105.8 | 86.8 | 96.4 | 107.2 |
| 60 | 110.5 | 92.8 | 103.2 | 87.2 | 91.6 | 104.7 | 110.6 | 92.8 | 103.9 | 87.2 | 92.3 | 104.6 |
| 61 | - | - | - | - | - | - | - | - | - | - | - | - |
| 62 | - | - | - | - | - | - | - | - | - | - | - | - |
| 63 | 109.9 | 102.6 | 105.4 | 90.5 | 103.1 | 106.4 | 109.8 | 103.1 | 105.9 | 89.8 | 103 | 106.9 |
| 64 | 112.6 | 98.7 | 100.5 | 91.9 | 98.1 | 98.5 | 113 | 99.5 | 101.2 | 91.7 | 98.4 | 98.9 |
| 65 | 113 | 95.2 | 105.9 | - | - | - | 114.1 | 96.3 | 106.8 | - | - | - |
| 66 | 111.7 | 102 | 107.2 | 89.6 | 101.9 | 106.5 | 113.4 | 104 | 107.1 | 89.5 | 103.7 | 107.4 |
| 67 | 108.8 | 98.1 | 106.3 | 87.4 | 95.7 | 105.6 | 108.3 | 99.1 | 106.9 | 86.6 | 96.8 | 106.5 |

Abbreviations: CT, computed tomography.

**Supplementary Table 5. Coordinates of the lowest electrode contact in dural puncture group**

| **Patient** | **Immediate CT** | | | | | | **One-month postoperative CT** | | | | | |
| --- | --- | --- | --- | --- | --- | --- | --- | --- | --- | --- | --- | --- |
|  | **Left hemisphere** | | | **Right hemisphere** | | | **Left hemisphere** | | | **Right hemisphere** | | |
|  | **X** | **Y** | **Z** | **X** | **Y** | **Z** | **X** | **Y** | **Z** | **X** | **Y** | **Z** |
| 1 | 111.2 | 95.7 | 100.1 | 91 | 96.2 | 99.8 | 112 | 96.3 | 101 | 91.6 | 97 | 99.8 |
| 2 | 115.8 | 104.7 | 115.4 | 91 | 103.2 | 116.4 | 115.4 | 105.2 | 115.9 | 90.1 | 103.7 | 116.5 |
| 3 | 87 | 98.7 | 115.5 | - | - | - | 86.6 | 99.6 | 115.3 | - | - | - |
| 4 | 108.3 | 97.6 | 107.9 | 84.9 | 100.3 | 106.3 | 109.4 | 99.6 | 106.7 | 85.2 | 101.7 | 107.2 |
| 5 | 112.9 | 104.6 | 89 | 87.9 | 104.2 | 87.8 | 113.6 | 105.5 | 89.7 | 87.8 | 104.9 | 88.6 |
| 6 | 111.7 | 92.9 | 106 | 89 | 91.8 | 108.4 | 112.6 | 93.8 | 106.2 | 89.5 | 92.8 | 108.9 |
| 7 | 110.7 | 98.2 | 109 | 88 | 99.2 | 110 | 110.7 | 99.3 | 109.7 | 87.9 | 99.8 | 110.7 |
| 8 | 107.4 | 103.9 | 119.4 | 83.8 | 102.3 | 120.5 | 107.9 | 103.7 | 119.7 | 84 | 103.2 | 120.7 |
| 9 | 108.5 | 92.6 | 107 | 87.8 | 92.7 | 105.3 | 109.8 | 94.1 | 105.9 | 88.3 | 93.5 | 105.5 |
| 10 | 111.1 | 95.5 | 112.1 | 89.4 | 95.3 | 112.8 | 111.6 | 95.8 | 112.5 | 89.5 | 96.5 | 112.7 |
| 11 | 112.5 | 97.7 | 106.1 | 88.1 | 97.7 | 105.3 | 113 | 98.3 | 106.9 | 87.5 | 98.3 | 106.1 |
| 12 | 109.6 | 97.6 | 107.5 | 88.5 | 94.8 | 109.1 | 109.5 | 98.4 | 108 | 88.7 | 95.5 | 109.6 |
| 13 | 108.2 | 93.9 | 106.7 | 88.1 | 95.2 | 105.6 | 108.5 | 94.2 | 107 | 87.8 | 96 | 105.7 |
| 14 | 110.7 | 93.9 | 113.3 | 90.8 | 92.5 | 112.5 | 111.8 | 93.7 | 113.2 | 91 | 92.6 | 112.5 |
| 15 | 106.4 | 96.6 | 108.1 | 85.7 | 96.5 | 109.8 | 106.5 | 97.5 | 108.4 | 85.9 | 97.4 | 110.2 |
| 16 | 110 | 95.9 | 106.7 | 87.2 | 96.2 | 105.9 | 109.5 | 96.4 | 107.3 | 86.6 | 96.5 | 106.4 |
| 17 | 109.9 | 91.8 | 95.6 | 90.2 | 93.4 | 95.1 | 109.9 | 92.6 | 96.1 | 89.5 | 93.9 | 95.7 |
| 18 | 109.9 | 91.9 | 95.5 | 90.3 | 93.2 | 95.3 | 109.9 | 92.5 | 96.1 | 89.6 | 94 | 95.5 |
| 19 | - | - | - | - | - | - | - | - | - | - | - | - |
| 20 | - | - | - | - | - | - | - | - | - | - | - | - |
| 21 | 109.4 | 107.1 | 119 | 85.8 | 106.9 | 120.7 | 110 | 107.7 | 119.6 | 85.7 | 108.1 | 120.9 |
| 22 | 110.6 | 99.6 | 103.5 | 88.4 | 98.3 | 103.2 | 110.3 | 100.6 | 104.3 | 88.1 | 99.3 | 103.8 |
| 23 | 109.2 | 98.8 | 102.9 | 84.7 | 98.8 | 100.8 | 108.7 | 98.9 | 103.1 | 83.8 | 98.9 | 101.2 |
| 24 | 112.2 | 91.4 | 105.9 | 89.2 | 92.6 | 105.6 | 112.1 | 92.7 | 106.7 | 88.7 | 93.7 | 106.6 |
| 25 | - | - | - | - | - | - | - | - | - | - | - | - |
| 26 | 113.9 | 102.1 | 107 | 91.5 | 104.4 | 104.4 | 113.5 | 103.3 | 107.7 | 91.6 | 104.1 | 106.5 |
| 27 | 113.2 | 98.5 | 107.4 | 87.8 | 97.8 | 106.3 | 113.4 | 99.1 | 107.7 | 87.5 | 98.6 | 106.9 |
| 28 | 85.6 | 97.3 | 97.4 | - | - | - | 85.1 | 98.6 | 97.7 | - | - | - |
| 29 | 88.8 | 100.7 | 103.7 | - | - | - | 88.9 | 101.3 | 104.2 | - | - | - |
| 30 | 87.2 | 94.2 | 104.4 | - | - | - | 87.5 | 94.8 | 104.8 | - | - | - |
| 31 | 86.3 | 94.3 | 110.7 | - | - | - | 86.5 | 94.6 | 111.6 | - | - | - |
| 32 | 110.6 | 95.2 | 105.6 | 89.8 | 94.8 | 104.3 | 111.2 | 95.5 | 106.1 | 89.9 | 95.3 | 104.7 |
| 33 | 111.4 | 101.4 | 107.6 | - | - | - | 112.1 | 101.8 | 108.2 | - | - | - |
| 34 | 107.9 | 100.6 | 103.2 | 88 | 101.1 | 102.1 | 108.3 | 101 | 103.7 | 87.9 | 101.7 | 102.5 |
| 35 | 106.9 | 99.2 | 108.7 | 84.1 | 99.2 | 109.7 | 107.6 | 99.6 | 109.2 | 83.5 | 99.3 | 109.9 |
| 36 | - | - | - | - | - | - | - | - | - | - | - | - |
| 37 | 112.8 | 92.8 | 105.9 | 92 | 90.8 | 107.9 | 112.5 | 92.8 | 107.6 | 92 | 91.3 | 108.4 |
| 38 | 104.8 | 98.8 | 110.4 | 83.9 | 101.1 | 110.1 | 105.2 | 99.9 | 110.7 | 83.8 | 102.2 | 110.3 |
| 39 | - | - | - | - | - | - | - | - | - | - | - | - |
| 40 | 109.6 | 102.8 | 97.9 | 85 | 102.3 | 100.4 | 109.8 | 103.6 | 98 | 84.8 | 102.6 | 101.1 |
| 41 | 110.7 | 96.7 | 118.5 | 87.6 | 95.7 | 119 | 111 | 97 | 118.9 | 87.9 | 95.8 | 119.3 |
| 42 | 116.3 | 95.3 | 103.1 | 90.6 | 95.4 | 105.7 | 116.2 | 96.2 | 103.7 | 90.6 | 97.1 | 106.1 |
| 43 | 112 | 96.1 | 125.2 | - | - | - | 112.2 | 96.1 | 125.7 | - | - | - |
| 44 | 87.1 | 92.6 | 102.5 | - | - | - | 87 | 93.6 | 103.4 | - | - | - |
| 45 | 106 | 97.8 | 106.9 | 84.1 | 95.9 | 105.8 | 106.7 | 98.7 | 105.8 | 83 | 96.2 | 106.6 |
| 46 | 113.2 | 96.7 | 101.2 | 89.1 | 97.3 | 100.4 | 112.5 | 97.1 | 101.9 | 89.5 | 97 | 101.4 |
| 47 | 112.6 | 98.3 | 103.6 | 88.8 | 99 | 102 | 112.8 | 99.2 | 103.6 | 88.8 | 99.3 | 102.6 |
| 48 | 112.6 | 97 | 105.1 | 87.5 | 94.8 | 107.8 | 112.7 | 97.9 | 105.5 | 86.8 | 94.6 | 109.3 |
| 49 | 110.5 | 99.7 | 92.9 | 88.7 | 100 | 93.1 | 110.8 | 100.2 | 93.7 | 88.5 | 100.7 | 93.9 |
| 50 | 87.9 | 93.8 | 106.9 | - | - | - | 88.3 | 95.5 | 107.5 | - | - | - |

Abbreviations: CT, computed tomography.


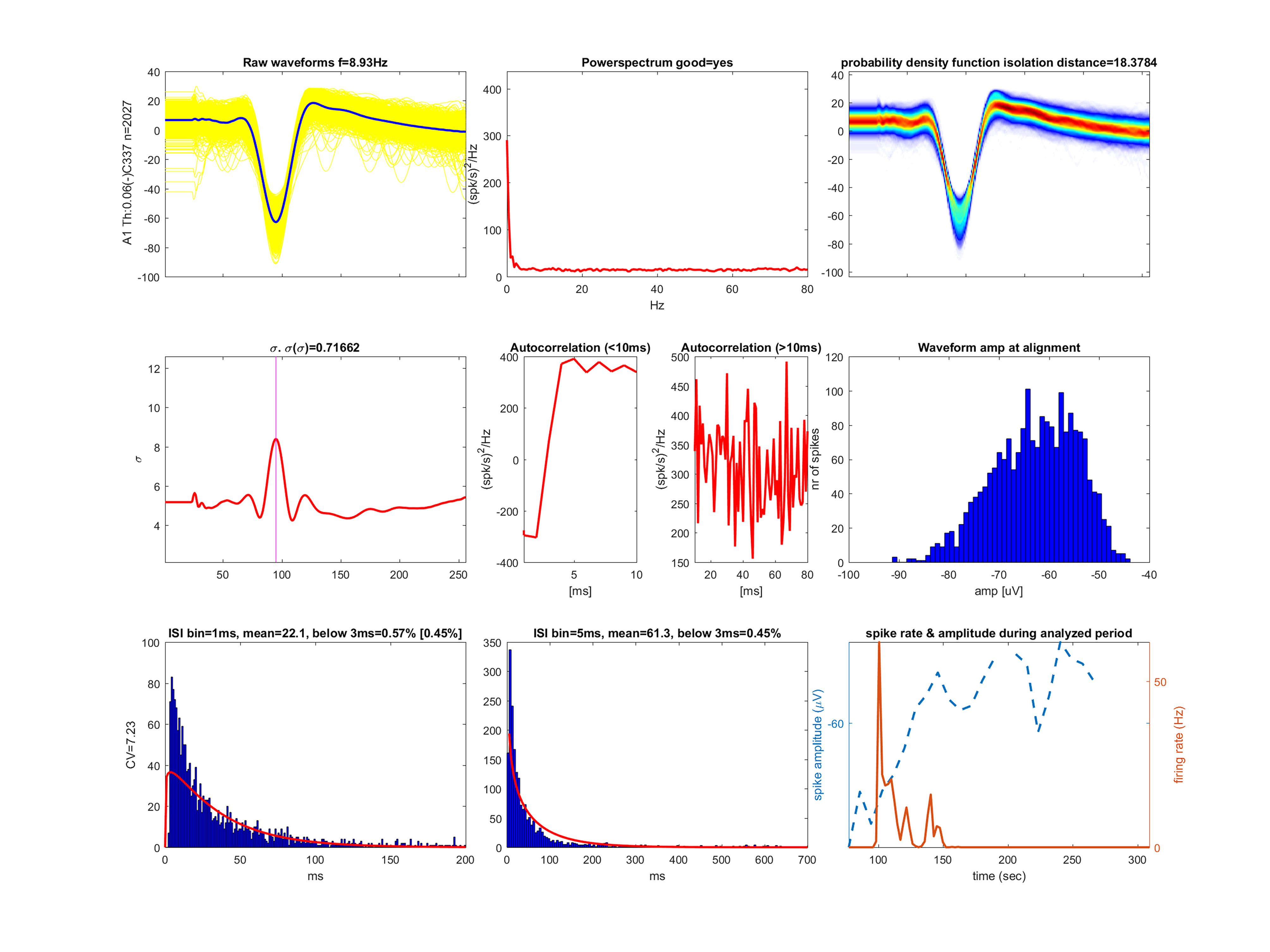


**Supplementary Figure 1.** Schematic diagram of a putative single neuron.
